# Supplementary material for: Deciphering the Genetic Variation: A Comparative Analysis of Parental and Attenuated Strains of the QXL87 Vaccine for Infectious Bronchitis
Source: Animals (Basel). 2024 Jun 13;14(12):1784. doi: 10.3390/ani14121784 (PMC11200882; doi:10.3390/ani14121784)
Supplement: Supplementary file 1 [file animals-14-01784-s001.zip › animals-3037131-supplementary.pdf]

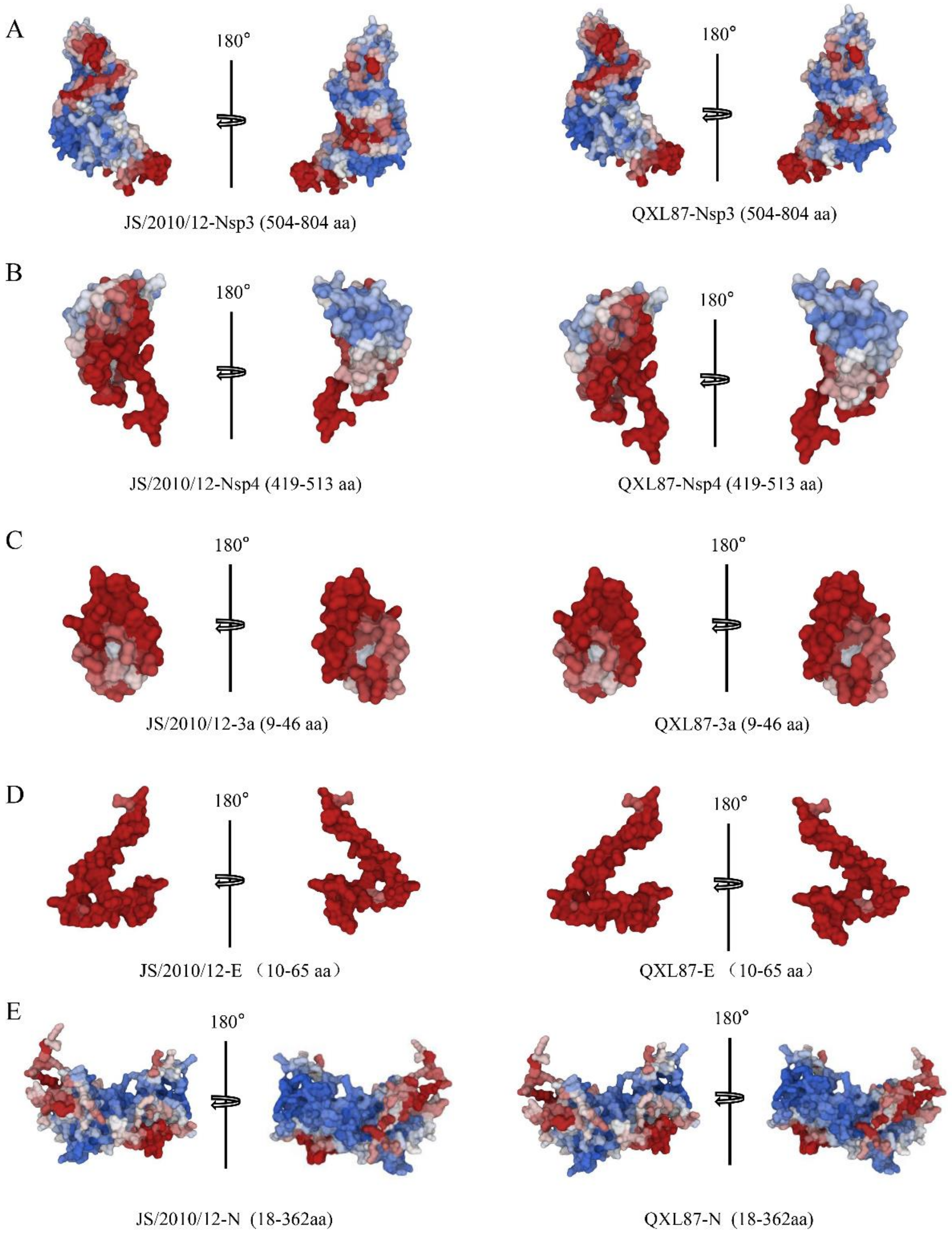

Figure S1. Prediction and comparison results of protein-protein binding sites in Nsp3 (A), Nsp4 (B), 3a (C), E (D), and N (E) protein of parental and attenuated strains. Color indicating binding propensity, from low (blue) to high (red).
